# Functional characterization and structural modeling of synthetic polyester-degrading hydrolases from *Thermomonospora curvata*

**DOI:** 10.1186/s13568-014-0044-9

**Published:** 2014-06-03

**Authors:** Ren Wei, Thorsten Oeser, Johannes Then, Nancy Kühn, Markus Barth, Juliane Schmidt, Wolfgang Zimmermann

**Affiliations:** 1 Department of Microbiology and Bioprocess Technology, Institute of Biochemistry, University of Leipzig, Johannisallee 21-23, Leipzig, D-04103, Germany

**Keywords:** Polyester hydrolase, Synthetic polyester, Polyethylene terephthalate (PET), Thermomonospora curvata

## Abstract

*Thermomonospora curvata* is a thermophilic actinomycete phylogenetically related to *Thermobifida fusca* that produces extracellular hydrolases capable of degrading synthetic polyesters. Analysis of the genome of *T. curvata* DSM43183 revealed two genes coding for putative polyester hydrolases Tcur1278 and Tcur0390 sharing 61% sequence identity with the *T. fusca* enzymes. Mature proteins of Tcur1278 and Tcur0390 were cloned and expressed in *Escherichia coli* TOP10. Tcur1278 and Tcur0390 exhibited an optimal reaction temperature against p-nitrophenyl butyrate at 60°C and 55°C, respectively. The optimal pH for both enzymes was determined at pH 8.5. Tcur1278 retained more than 80% and Tcur0390 less than 10% of their initial activity following incubation for 60 min at 55°C. Tcur0390 showed a higher hydrolytic activity against poly(ε-caprolactone) and polyethylene terephthalate (PET) nanoparticles compared to Tcur1278 at reaction temperatures up to 50°C. At 55°C and 60°C, hydrolytic activity against PET nanoparticles was only detected with Tcur1278. *In silico* modeling of the polyester hydrolases and docking with a model substrate composed of two repeating units of PET revealed the typical fold of α/β serine hydrolases with an exposed catalytic triad. Molecular dynamics simulations confirmed the superior thermal stability of Tcur1278 considered as the main reason for its higher hydrolytic activity on PET.

## Introduction

The widespread use of synthetic polyesters such as polyethylene terephthalate (PET) in industry and daily life has resulted in serious environmental pollution over the last decades. However, the recycling of PET by chemical methods performed under extreme temperature and pH conditions is an energy-consuming process (Paszun and Spychaj [1997]). Recently, alternative processes using biocatalysis have been proposed for recycling and surface functionalization of PET (Müller et al. [2005]; Zimmermann and Billig [2011]). Microbial enzymes capable of degrading PET have been previously described from various fungal (Egmond and de Vlieg [2000]; Alisch et al. [2004]; Alisch-Mark et al. [2006]; Nimchua et al. [2007]; Ronkvist et al. [2009]) and bacterial (Müller et al. [2005]; Eberl et al. [2009]; Herrero Acero et al. [2011]; Ribitsch et al. [2012a]; Ribitsch et al. [2012b]; Sulaiman et al. [2012]; Kitadokoro et al. [2012]; Chen et al. [2010]; Oeser et al. [2010]) sources. Enzymes with high PET-hydrolyzing activity are mostly extracellular proteins secreted by thermophilic microorganisms such as *Thermomyces insolens* (Ronkvist et al. [2009]) and several *Thermobifida* species (Müller et al. [2005]; Eberl et al. [2009]; Herrero Acero et al. [2011]; Ribitsch et al. [2012a]; Ribitsch et al. [2012b]; Kitadokoro et al. [2012]; Chen et al. [2010]; Oeser et al. [2010]). The biodegradability of PET by these enzymes has been shown to strongly depend on the flexibility of polymer chains that is directly influenced by the hydrolysis reaction temperatures (Ronkvist et al. [2009]; Wei et al. [2013]).

*Thermomonospora curvata* DSM 43183, a facultative aerobic thermophilic actinomycete, has been isolated from composts containing plant materials (Henssen [1957]; Henssen and Schnepf [1967]; Chertkov et al. [2011]). The optimal growth temperature of *T. curvata* is 50°C (Henssen and Schnepf [1967]) at a wide range of pH from 7.5 to 11 (Chertkov et al. [2011]). Weak growth of *T. curvata* has been also observed at higher temperatures up to 65°C (Henssen and Schnepf [1967]). The phylogenetic analysis of *T. curvata* revealed a distant relationship to other thermophilic actinomycetes isolated from a similar habitat including *Thermobifida fusca* and *Thermobifida alba*, as indicated by a lower level of 16S rRNA sequence similarity between 89% and 90% (Henssen [1957]; Zhang et al. [1998]; Chertkov et al. [2011]). Several of these bacteria have been shown to express extracellular enzymes with polyester-hydrolyzing activity (Kleeberg et al. [1998]; Alisch et al. [2004]; Herrero Acero et al. [2011]; Thumarat et al. [2012]; Ribitsch et al. [2012b]).

In this study, we report the identification of two genes coding for the polyester hydrolases Tcur1278 and Tcur0390 by genome mining of *T. curvata* DSM43183 (Chertkov et al. [2011]), the characterization of their catalytic properties and thermal stability, as well as the modeling and analysis of their three-dimensional structures.

## Materials and methods

### Cloning, expression and purification of Tcur1278 and Tcur0390

The genes encoding Tcur1278 and Tcur0390 without the Gram-positive secretion signal peptides were selected from the annotated genome sequences of *T. curvata* DSM43183 (Chertkov et al. [2011]). Synthetic gene constructs with adapted codon usage to *E. coli* (Geneart GmbH, Regensburg, Germany) for Tcur1278 [EMBL: HG939554] and Tcur0390 [EMBL: HG939555] were applied for direct cloning into the pBAD TOPO expression vector (Invitrogen, Life Technologies, Carlsbad, USA). The recombinant expression of *T. curvata* hydrolases was carried out in One Shot *E. coli* TOP10 (Invitrogen) at room temperature for 14 h in lysogeny broth (LB) containing 0.2% (m/v) of L-arabinose as inducer as described previously (Oeser et al. [2010]). Bacterial cells were harvested by centrifugation and resuspended in a lysis buffer containing 50 mM phosphate (pH 8) and 300 mM NaCl. After sonication, the soluble cell extracts were subjected to immobilized metal ion affinity chromatography (IMAC) using Ni-NTA columns (Qiagen, Hilden, Germany). The protein elutions containing the recombinant hydrolases were separated by SDS PAGE and analyzed by esterase activity-staining with 1-naphthyl acetate and Fast Red dye (Sztajer et al. [1992]) as well as by staining with Coomassie Brilliant Blue.

### Determination of esterase activity

Esterase activity was determined with p-nitrophenyl butyrate (pNPB) as a substrate in a microplate format (BioTek PowerWave XS, BioTek Instruments Inc., Winooski, USA) (Billig et al. [2010]). To avoid the adsorption of proteins to the plastic vials, the dilution was carried out in the presence of 15% poly(ethylene glycol) (PEG_6000_, Sigma-Aldrich Co., St. Louis, USA) in Davies buffer (Davies [1959]) between pH 6.5 and 9.5 or in 100 mM Tris-HCl. One unit of esterase activity was defined as the amount of enzyme required to hydrolyze 1 μmol pNPB per min (Alisch et al. [2004]). To investigate their thermal stability, 250 μg/mL of enzymes were incubated in 100 mM Tris buffer (pH 8.5) at 50°C, 55°C and 60°C for up to 1 h. Residual esterase activity against pNPB was determined at 25°C in triplicate. The Michaelis-Menten kinetic constants for the hydrolysis of pNPB by Tcur1278 and Tcur0390 were determined at 25°C and pH 8.5.

### Enzymatic hydrolysis of polyester nanoparticles

The enzymatic hydrolysis of polyesters was analyzed by monitoring the change of turbidity of a polyester nanoparticle suspension at 600 nm (Wei et al. [2013]). Poly(ε-caprolactone) (PCL) and PET nanoparticles were prepared by a precipitation and solvent evaporation technique from amorphous PCL (Sigma-Aldrich Co., St. Louis, USA) and low-crystallinity PET film (Goodfellow GmbH, Bad Nauheim, Germany) dissolved in acetone and 1,1,1,3,3,3-hexafluoro-2-propanol, respectively. The enzymatic hydrolysis of PCL was performed in a microplate format (BioTek PowerWave XS) at 49°C with 0.22 mg/mL of PCL nanoparticles in each well, whereas the enzymatic hydrolysis of PET was performed at 50°C to 60°C in cuvettes containing 0.25 mg/mL of PET nanoparticles immobilized in 0.9% agarose gel. The change of turbidity was monitored over an incubation period of 15 min at 1 min intervals for PCL hydrolysis, whereas PET hydrolysis was determined for 60 min at 5 min intervals. The initial degradation rates were defined as the square roots of turbidity decrease during the initial linear phase of the hydrolysis, and plotted as a function of enzyme concentration using a kinetic model (Eq. 1) modified from Wei et al. ([2013])

(1) $$\frac{d\left(\sqrt{\tau }\right)}{\mathrm{dt}}=\frac{k_{\tau }K_{A}\left[E\right]}{1+K_{A}\left[E\right]}$$

where *τ* is the turbidity of a nanoparticle suspension, *t*, the reaction time, *k*_*τ*_, the hydrolysis rate constant based on the turbidity change, *K*_*A*_, the adsorption equilibrium constant, and [*E*], the enzyme concentration.

### Homology modeling and molecular docking

Homology modeling of *T. curvata* polyester hydrolases was carried out using the Phyre2 web server (Kelley and Sternberg [2009]) based on the crystal structure of *T. alba* AHK 119 (Est119, PDB ID: 3VIS) (Kitadokoro et al. [2012]). The sequence identity of *T. curvata* polyester hydrolases with the corresponding template structure is summarized in Table 1 and Additional file 1: Figure S1.

**Table 1:** **Sequence identity (in percent, upper right part) and the root-mean-square deviation (RMSD) of C**_**α**_**atoms (in** Å, **lower left part) of*T. curvata*polyester hydrolases in comparison with the crystal structure of homologous*T. alba*Est119 (PDB ID: 3VIS)**

	**Identity (%)**		
**RMSD (Å)**	**Tcur1278**	**Tcur0390**	***T. alba*** **Est119**
**Tcur1278**		82.2	61.7
**Tcur0390**	0.11		61.7
***T. alba*** **Est119**	0.85	0.83	

The molecular docking program GOLD version 5.1 (Cambridge Crystallographic Data Centre, Cambridge, UK) (Jones et al. [1997]) was used to study the substrate-binding pocket of *T. curvata* polyester hydrolases. The polyester model substrate 2PET composed of 2 repeating units of PET (*bis* 2-hydroxyethyl terephthalate, BHET) was constructed with the software MOE (Chemical Computing Group, Montreal, Canada). The central ester bond of 2PET was constrained in the oxyanion hole composed of the main chain NH groups of amino acid residues F62 and M131 with the correct orientation to form a tetrahedral intermediate based on the catalytic mechanism of ester hydrolases (Jaeger et al. [1999]). The other atoms of 2PET were allowed to be flexible for a conformation to be docked to the rigid protein structural model by a genetic algorithm (Jones et al. [1997]). Based on the default scoring function of GOLD, the top-ranked productive docking conformations in accordance with the catalytic mechanism of ester hydrolases (Jaeger et al. [1999]) were selected for the illustrations generated by the MOE software.

### Molecular dynamics simulations

The molecular dynamics (MD) simulations were carried out using GROMACS 4.6 (Groningen University, The Netherlands) (Hess et al. [2008]) in the Amber99SB force field (Hornak et al. [2006]) in explicit solvent. Protein structural models of both *T. curvata* polyester hydrolases were centered in a cube with a distance of ≥1.0 nm from each edge as the starting structures. The steepest descent method was applied to perform energy minimization until a maximum force (F_max_) of less than 1000 kJ/mol/nm was reached. The system was equilibrated for 100 ps by a position-restrained simulation at the desired temperatures in the isothermal-isobaric (NPT) ensemble. The isotropic pressure coupling using the Berendsen algorithm was applied with a reference pressure of 1.0 bar (Berendsen et al. [1984]). For each protein structure, three independent simulations were performed under equilibration conditions for 50 ns in 2 fs steps at 298 K (25°C) and 353 K (80°C), respectively. To analyze the thermal stability of the polyester hydrolases, the time course of the root-mean-square deviation (RMSD) of backbone structures and the root-mean-square fluctuation (RMSF) of C_α_ atoms of each amino acid residue over the complete 50 ns simulation were calculated using GROMACS 4.6 (Hess et al. [2008]).

## Results

### Cloning, expression and purification of Tcur1278 and Tcur0390

Synthetic genes encoding Tcur1278 and Tcur0390 were amplified in the pBAD-TOPO expression vector (Invitrogen) for recombinant expression in One Shot *E. coli* TOP10 (Invitrogen). Following an expression period of 14 h at 25°C and the subsequent IMAC purification, 2.5 mg of Tcur1278 and 2.9 mg of Tcur0390 were obtained from a 500 mL culture with a specific activity of 3.0 U/mg and 17.9 U/mg against pNPB, respectively. By SDS PAGE analysis, both *T. curvata* hydrolases were obtained as single bands with esterase activity against 1-naphthyl acetate, corresponding to an apparent molecular mass of approximately 35 kDa (Additional file 1: Figure S2).

### Effect of pH and temperature on the hydrolytic activity of Tcur1278 and Tcur0390

**Figure 1:**
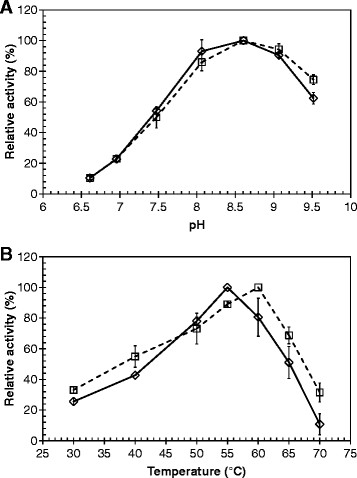
**Effects of pH and temperature on the hydrolytic activity of Tcur1278 and Tcur0390.** Activities of Tcur1278 and Tcur0390 against pNPB at different **(A)** pH and **(B)** reaction temperature conditions are shown as broken and solid lines, respectively. Error bars indicate the standard deviation of three determinations.

The effect of pH on the hydrolytic activity of Tcur1278 and Tcur0390 was investigated against pNPB in a pH range from 6.5 to 9.5 (Figure 1A). Both enzymes displayed an optimal pH at pH 8.5 and still retained more than 60% of their maximum activity at pH 9.5.

The effect of temperature on the hydrolytic activity of both enzymes was assayed against pNPB in a temperature range from 30°C to 70°C (Figure 1B). Tcur1278 and Tcur0390 showed an optimal temperature at 60°C and 55°C, respectively.

### Thermal stability of Tcur1278 and Tcur0390

**Figure 2:**
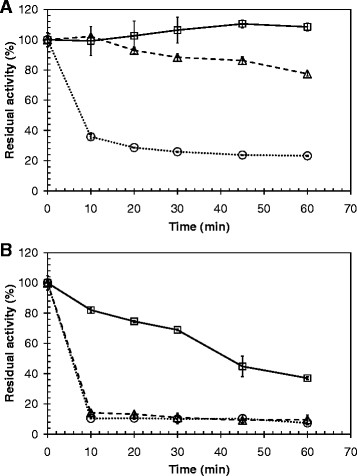
**Thermal stability performance of Tcur1278 and Tcur0390.** The residual hydrolytic activity was determined with **(A)** Tcur1278 and **(B)** Tcur0390 against pNPB over a period of 1 h at 50°C (solid line), 55°C (broken line) and 60°C (dotted line). Error bars indicate the standard deviation of three determinations.

The stability of Tcur1278 and Tcur0390 at 50°C, 55°C and 60°C was investigated at pH 8.5 over a period of 60 min by monitoring the residual activities against pNPB (Figure 2A-B). Tcur1278 showed a higher thermal stability compared to Tcur0390 retaining more than 80% of its initial activity following incubation for 60 min at 50°C and 55°C. At 60°C, approximately 65% loss of its initial activity was detected following incubation for 10 min. In contrast, Tcur0390 showed a residual activity of only 40% following incubation for 60 min at 50°C and of 15% following incubation for 10 min at 55°C and 60°C.

### Kinetic analysis of the hydrolysis of pNPB by Tcur1278 and Tcur0390

Based on the Michaelis-Menten kinetic model, Tcur0390 revealed an almost 6-fold higher *k*_*cat*_ and no significantly lower *K*_m_ than Tcur1278 for pNPB hydrolysis indicating a higher hydrolytic activity of Tcur0390 against the soluble pNPB compared to Tcur1278 (Table 2).

**Table 2:** **Kinetic parameters for pNPB hydrolysis by Tcur1278 and Tcur0390 at 25°C and pH 8.5**

	**Tcur1278**	**Tcur0390**
***K***_**m**_**(μM)**	88.8 ± 12.8	83.1 ± 11.1
***k***_**cat**_**(1/s)**	2.3 ± 0.1	12.4 ± 0.4

### Kinetic analysis of the hydrolysis of PCL nanoparticles by Tcur1278 and Tcur0390

**Figure 3:**
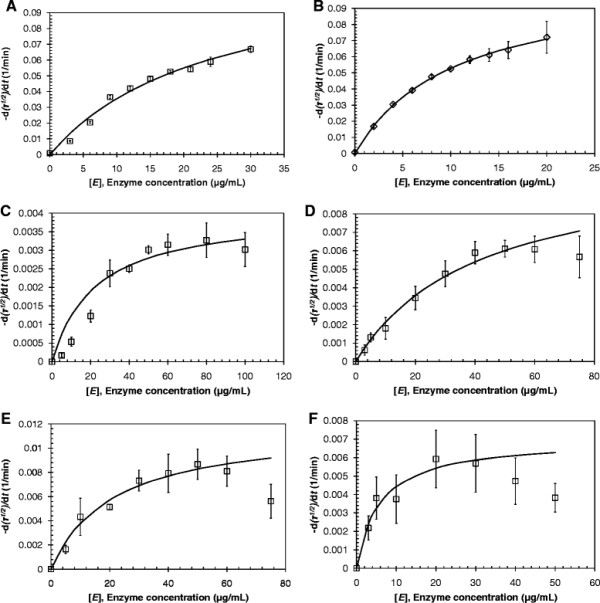
**Decomposition of polyester nanoparticles by*T. curvata*hydrolases.** PCL hydrolysis by **(A)** Tcur1278 and **(B)** Tcur0390 at 49°C; PET hydrolysis by Tcur1278 at **(C)** 50°C, **(D)** 55°C and **(E)** 60°C, and by Tcur0390 at **(F)** 50°C. The initial rates of the square roots of turbidity decrease are plotted as a function of enzyme concentration (squares and diamonds). Error bars represent the standard deviation of duplicate determinations. Fitted data (solid lines) according to Eq. (1) are also shown.

A PCL nanoparticle suspension with a concentration of 0.22 mg/mL was completely hydrolyzed following incubation for 15 min at 49°C with 20 μg/mL of Tcur0390 or 30 μg/mL of Tcur1278 (data not shown). The kinetic analysis of the hydrolysis of PCL nanoparticles was therefore performed at enzyme concentrations up to 20 μg/mL and 30 μg/mL for Tcur0390 and Tcur1278, respectively. The hydrolysis rates of PCL nanoparticles calculated from the square roots of turbidity decrease are shown as a function of enzyme concentration (Figure 3A-B). By fitting the experimental data to Eq. (1), the kinetic constants for the PCL nanoparticle hydrolysis by the two enzymes were determined (Table 3). Compared to Tcur1278, Tcur0390 showed a 2.3-fold higher adsorption equilibrium constant (*K*_*A*_) and no significantly higher hydrolysis rate constant (*k*_*τ*_).

**Table 3:** **Kinetic parameters for polyester nanoparticle hydrolysis by Tcur1278 and Tcur0390 at 49**°**C to 60°C and pH 8.5**

**Polyester nanoparticles**		**PCL**	**PET**		
**Temperature (°C)**		**49**	**50**	**55**	**60**
**Tcur1278**	***K***_**A**_**(mL/mg)**	41.1 ± 4.5	44.4 ± 8.6	24.3 ± 5.3	46.9 ± 12.1
	***k***_***τ***_**(10**^**-3**^**/min)**	122.2 ± 11.9	4.1 ± 0.5	11.0 ± 1.2	11.8 ± 1.2
**Tcur0390**	***K***_**A**_**(mL/mg)**	96.0 ± 9.8	172.7 ± 30.7	n. d.	n. d.
	***k***_***τ***_**(10**^**-3**^**/min)**	108.3 ± 5.7	7.0 ± 0.8	n. d.	n. d.

n. d. = not detectable.

### Kinetic analysis of the hydrolysis of PET nanoparticles by Tcur1278 and Tcur0390

The enzymatic hydrolysis of PET nanoparticles by Tcur1278 and Tcur0390 was investigated at pH 8.5 and temperatures of 50°C, 55°C and 60°C (Figure 3C-F). Due to the lower thermal stability of Tcur0390 (Figure 2B), a hydrolytic activity at 55°C and 60°C was not detected. At 50°C, a maximum hydrolysis rate ($d\left(\sqrt{\tau }\right)/\mathrm{dt}$) of 3.3 × 10^-3^ min^-1^ and 5.9 × 10^-3^ min^-1^ was determined with 80 μg/mL of Tcur1278 and 20 μg/mL of Tcur0390, respectively. With 50 μg/mL of Tcur1278, the hydrolysis rate was increased 1.8-fold at 55°C and 2.6-fold at 60°C. Higher enzyme concentrations exceeding the amount required for the maximum reaction rate resulted in lower hydrolysis rates. This effect has also been observed in the hydrolysis of PET nanoparticles by TfCut2, a polyester hydrolase from *T. fusca*, and has been attributed to the adsorption of catalytically inactive enzyme in excess to the monolayer coverage of the PET surface (Wei et al. [2013]).

Table 3 summarizes the kinetic constants for the enzymatic PET nanoparticle hydrolysis by fitting the experimental data to Eq. (1). Compared to Tcur1278, a 1.7-fold higher *k*_*τ*_ and a 3.9-fold higher *K*_*A*_ were obtained with Tcur0390 at 50°C. With Tcur1278, the highest values of both kinetic constants were determined at 60°C.

### *In silico* modeling of Tcur1278 and Tcur0390

Structural models of Tcur1278 and Tcur0390 were generated on the basis of the crystal structure of Est119 from *T. alba* AHK119 (PDB ID: 3VIS) (Kitadokoro et al. [2012]). Homology models of *T. curvata* polyester hydrolases revealed a typical α/β hydrolase fold (Ollis et al. [1992]; Carr and Ollis [2009]) with low RMSD values of C_α_ atomic coordinates of less than 1 Å in comparison with the template crystal structure (Table 1).

**Figure 4:**
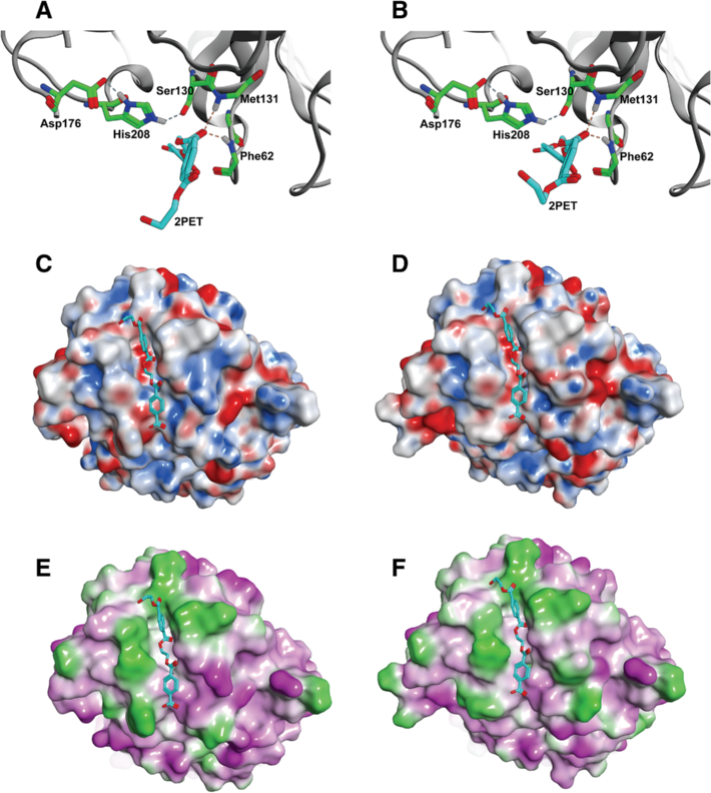
**Structural modeling of Tcur1278 and Tcur0390 polyester hydrolases.** Homology modeling was performed with the Phyre2 web server (Kelley and Sternberg [2009]). The catalytic triad of **(A)** Tcur1278 and **(B)** Tcur0390 is formed by S130, D176 and H208. The 2PET model substrate was docked using GOLD 5.1 with its central ester bond constrained between 2.7 and 3.1 Å in the oxyanion hole formed by the main chain NH groups of F62 and M131 (broken yellow lines). The hydrogen bonds stabilizing the tetrahedral intermediate formed during the catalytic reaction are shown as broken lines in blue. The backbone structures are shown as gray cartoons. The electrostatic surface properties of Tcur1278 **(C)** and Tcur0390 **(D)** are shown with negatively charged residues in red, positively charged residues in blue and neutral residues in white/gray, respectively. The lipophilic surface properties of Tcur1278 **(E)** and Tcur0390 **(F)** are shown with hydrophilic residues in pink and hydrophobic residues in bright green, respectively. The docked 2PET model substrate is shown in cyan.

Similar to TfCut2 from *T. fusca* KW3 (Wei [2011]; Herrero Acero et al. [2011]), the catalytic triad of *T. curvata* polyester hydrolases formed by S130, D176 and H208 was found to be exposed to the solvent (Figure 4A-B). By docking of the 2PET model substrate, the substrate-binding pocket could be identified as a large groove on the surface of Tcur1278 and Tcur0390 (Figure 4C-F). The negative charge buried in this major groove was contributed by the deprotonated S130 (Figure 4C-D). As shown in Figure 4C-F, Tcur1278 and Tcur0390 displayed similar surface properties in the vicinity of the active site with extended hydrophobic regions around the substrate-binding groove.

### Molecular dynamics simulations of Tcur1278 and Tcur0390

**Figure 5:**
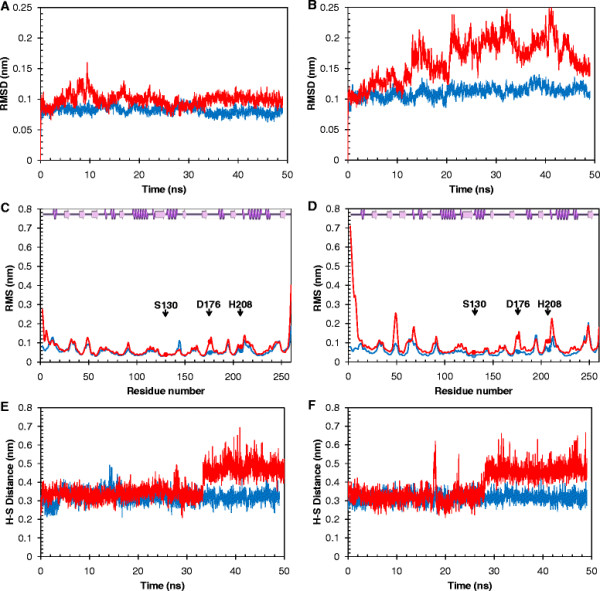
**Molecular dynamics simulation of (A, C, E) Tcur1278 and (B, D, F) Tcur0390 polyester hydrolases. (A, B)** Time courses of backbone RMSD changes during a simulation for 50 ns at 298 K (blue) and 353 K (red). **(C, D)** RMSF of C_α_ atoms per amino acid residue during a simulation for 50 ns at 298 K (blue) and 353 K (red). The purple spirals and arrows at the top of the RMSF graphs indicate α-helices and β-sheets, respectively. The catalytic triad residues are shown as solid spheres. **(E, F)** The distance of the catalytic H208 and S130 (H-S) during a simulation for 50 ns at 298 K (blue) and 353 K (red). For a clearer view, single simulation data from three simulations are shown.

The overall C_α_ RMSD values for *T. curvata* polyester hydrolases obtained by MD simulations at 298 K and 353 K are shown as a function of simulation time (Figure 5A-B). In all simulations, the RMSD values for both proteins stabilized rapidly within 0.02 ns. At 298 K, the RMSD for Tcur1278 showed values below 0.1 nm, slightly lower than the corresponding values for Tcur0390. At 353 K, the RMSD values for Tcur0390 fluctuated more strongly compared to Tcur1278. This effect was most pronounced after 15 ns simulation time. The RMSD values for Tcur0390 were almost doubled at 353 K compared to those obtained at 298 K. In contrast, Tcur1278 exhibited only a slight increase in backbone structure deviations at 353 K further confirming its superior thermal stability properties.

The corresponding RMSF plots revealed the flexibility profiles for the complete protein sequence (Figure 5C-D). Both the enzymes displayed a flexible N-terminus with the highest deviation of C_α_ atoms contributed mainly by a short helical part of the molecule (12-17) embedded in loop structures (2-11, 18-23). The high RMSF observed in this part of the protein also affected the neighboring beta-sheet structure (24-30) and furthermore the whole enzyme. Compared to Tcur1278, Tcur0390 showed generally a larger difference in the flexibility profiles obtained by the MD simulations performed at temperatures from 298 K to 353 K. A significant increase of the RMSF values at higher temperatures was observed in the neighborhood of D176 (172-180) with both enzymes. This resulted also in a higher flexibility of H208 at 353 K due to its interaction with D176 according to the catalytic mechanism of ester hydrolases (Jaeger et al. [1999]). As a consequence, the distance between the catalytic H208 and S130 (H-S) increased from 0.3 nm to 0.5 nm after 34 ns and 28 ns of the MD simulation at 353 K with Tcur1278 and Tcur0390, respectively (Figure 5E-F). Compared to the RMSD plot shown in Figure 5A that suggested a relatively stable backbone structure of Tcur1278 during the complete 50 ns simulation at 353 K, the permanent change of the H-S distance occurred at an earlier stage prior to the denaturation of its other temperature-labile parts.

## Discussion

Bacterial polyester hydrolases have been previously described mainly from thermophilic *Thermobifida* species (Kleeberg et al. [1998]; Alisch et al. [2004]; Herrero Acero et al. [2011]; Thumarat et al. [2012]; Ribitsch et al. [2012b]; Oeser et al. [2010]). *T. curvata* is a phylogenetically related actinomycete that has been isolated from a similar habitat (Henssen [1957]; Zhang et al. [1998]; Chertkov et al. [2011]). By genome mining of *T. curvata* DSM 43183 (Chertkov et al. [2011]), we identified two genes encoding the proteins Tcur1278 and Tcur0390 with sequences similar to TfCut2 from *T. fusca* KW3 (Wei [2011]; Herrero Acero et al. [2011]). As shown in the protein sequence alignment (Simossis and Heringa [2005]), Tcur1278 and Tcur0390 share a sequence identity of about 82% and both enzymes show a sequence identity of about 61% with TfCut2 (Additional file 1: Figure S1).

Codon-optimized genes of Tcur1278 and Tcur0390 were synthesized for cloning and recombinant expression in *E. coli*. When the pET-20b(+) vector (Novagen) was used for the recombinant expression of the complete proteins of Tcur1278 and Tcur0390 in *E. coli* BL21(DE3), no active proteins could be detected suggesting an interference of the original Gram-positive signal peptides with the recombinant system. With the pBAD expression vector and *E. coli* TOP10 (Invitrogen) for shorter mature proteins, both *T. curvata* polyester hydrolases could be expressed as active enzymes fused with a C-terminal His-tag and purified by affinity chromatography (Additional file 1: Figure S2).

Similar to homologous polyester hydrolases from *T. fusca* (Chen et al. [2008]; Wei [2011]; Herrero Acero et al. [2011]), Tcur1278 and Tcur0390 showed their highest activity against pNPB between pH 8 and pH 9 in a temperature range from 50°C to 60°C (Figure 1). Compared to Tcur1278, Tcur0390 revealed a significantly higher hydrolytic activity against both soluble (pNPB) and insoluble substrates (polyester nanoparticles) at reaction temperatures up to 50°C (Figure 3, Tables 2 and 3). This higher hydrolytic activity could be attributed to the stronger substrate affinity of Tcur0390 (Table 3). Molecular docking experiments with the model substrate 2PET to the structural models of *T. curvata* polyester hydrolases confirmed the presence of extended hydrophobic regions in close vicinity to the catalytic triad (Figure 4E-F). The hydrophobic character of the regions near the substrate-binding groove may facilitate the binding of hydrophobic polymeric substrates. Compared to Tcur1278, the hydrophobic properties were more pronounced in Tcur0390 and may account for its observed higher substrate affinity (Figure 4E-F). This result is confirming earlier observations that more hydrophobic and less charged amino acid residues clustered in the neighborhood of the substrate-binding groove of Thc_Cut1 compared to Thc_Cut2 and a concomitantly higher hydrolytic activity of the former isoenzyme from *T. cellulosilytica* (Herrero Acero et al. [2011]; Herrero Acero et al. [2013]).

The optimal temperatures for pNPB hydrolysis by Tcur1278 and Tcur0390 were 60°C and 55°C, respectively (Figure 1B). However, both enzymes showed poor thermal stability at their optimal temperature, as indicated by an irreversible loss of more than 65% of their initial activities following incubation for 10 min (Figure 2A-B). Tcur1278 maintained its maximum activity against PET nanoparticles for approximately 15 min at 60°C (data not shown). This suggests that the thermal stability of Tcur1278 was improved in the presence of the insoluble polymeric substrate. In contrast, a significant improvement of the thermal stability was not detected with Tcur0390 in the presence of PET nanoparticles. The backbone RMSD plots obtained by MD simulations also indicated a more rigid structure of Tcur1278 thus verifying its superior thermal stability compared to Tcur0390 (Figure 5A-B). The RMSF profiles that describe the deviation of C_α_ atoms of single amino acid residues from the averaged position over the simulation period showed highly flexible regions clustered in the neighborhood of the catalytic residues H208 and D176 (Figure 5C-D). These regions may enable some induced fit motions necessary for the catalytic reaction at the active site. A comparison of the backbone RMSD plots (Figure 5A) and the H-S distance of Tcur1278 (Figure 5E) over the complete MD simulation period indicated that the exposed flexible catalytic triad was also prone to local unfolding prior to the denaturation of the overall structure. By contrast, the permanent increase of the H-S distance in Tcur0390 was accompanied by the unfolding of the overall structure and occurred at an earlier stage of MD simulations compared to Tcur1278 (Figure 5B, F).

A reaction temperature close to the glass transition temperature of PET at approximately 75°C (Alves et al. [2002]) is required for an optimal performance of the enzymatic hydrolysis due to the restricted mobility of polymer chains at temperatures below (Marten et al. [2003,2005]; Herzog et al. [2006]; Ronkvist et al. [2009]; Wei et al. [2013]). The thermal stability of Tcur1278 needs therefore be further improved for an efficient degradation of PET. Protein engineering in regions near the catalytic triad as well as at the flexible N-terminus could be a useful approach for further optimizations to overcome the limited thermal stability of these polyester hydrolases.

In summary, the polyester hydrolases Tcur1278 and Tcur0390 from *T. curvata* have been shown to exhibit catalytic and structural features similar to enzymes from *T. fusca* and *T. cellulosilytica*. The comparison of the catalytic characteristics of Tcur1278 and Tcur0390 revealed a correlation between their hydrolytic activity and their surface properties in the vicinity of the catalytic triad. However, a comparison of the thermal stability of the two enzymes provided evidence that their ability to hydrolyze PET is predominately limited by their stability at higher reaction temperatures.

## Competing interests

The authors declare that they have no competing interests.

## Authors’ contributions

RW and NK carried out the recombinant cloning of genes coding for the polyester hydrolases. MB and JS participated in the expression and purification of the recombinant enzymes. TO and RW carried out the biochemical characterization of the polyester hydrolases. JT performed the molecular dynamics simulations. RW and TO analyzed the experimental and simulation data and prepared the manuscript. WZ conceived the study and contributed to manuscript writing. All authors read and approved the final manuscript.

## Additional file

## Supplementary Material

Additional file 1: Figure S1. Alignment of the mature protein sequences of Tcur1278, Tcur0390, TfCut2 and Est119 polyester hydrolases. The regions of similarity of individual amino acid residues are indicated with colors from blue, unconserved to red, conserved. The multiple sequence alignment was performed with the PRALINE web server (Simossis and Heringa [2005]). **Figure S2.** SDS PAGE analysis of Tcur0390 (lanes 1-2) and Tcur1278 (lanes 3-4). 10 μg of crude cell lysate (1, 3) and eluate obtained after IMAC purification (2, 4) were loaded in each lane; protein size markers (M). The gel was first stained with Fast Red dye for esterase activity against 1-naphthyl acetate (purple bands) followed by staining with Coomassie Brilliant Blue (blue bands). Click here for file
