# Supplementary material for: Functional characterization and structural modeling of synthetic polyester-degrading hydrolases from Thermomonospora curvata
Source: AMB Express. 2014 Jun 3;4:44. doi: 10.1186/s13568-014-0044-9 (PMC4231364; doi:10.1186/s13568-014-0044-9)
Supplement: Additional file 1: Figure S1. — Alignment of the mature protein sequences of Tcur1278, Tcur0390, TfCut2 and Est119 polyester hydrolases. The regions of similarity of individual amino acid residues are indicated with colors from blue, unconserved to red, conserved. The multiple sequence alignment was performed with the PRALINE web server (Simossis and Heringa [2005]). Figure S2. SDS PAGE analysis of Tcur0390 (lanes 1-2) and Tcur1278 (lanes 3-4). 10 μg of crude cell lysate (1, 3) and eluate obtained after IMAC purification (2, 4) were loaded in each lane; protein size markers (M). The gel was first stained with Fast Red dye for esterase activity against 1-naphthyl acetate (purple bands) followed by staining with Coomassie Brilliant Blue (blue bands). [file s13568-014-0044-9-S1.pdf]

Functional characterization and structural modeling of synthetic polyester-degrading hydrolases from *Thermomonospora curvata*

Ren Wei, Thorsten Oeser, Johannes Then, Nancy Kühn, Markus Barth, Juliane Schmidt, Wolfgang Zimmermann*

Department of Microbiology and Bioprocess Technology, Institute of Biochemistry, University of Leipzig, Johannisallee 21-23, D-04103 Leipzig, Germany

* Corresponding author.

Tel.: +49 341 97-36781; fax: +49 341 +49 341 97-36798.

E-mail address: wolfgang.zimmermann@uni-leipzig.de.

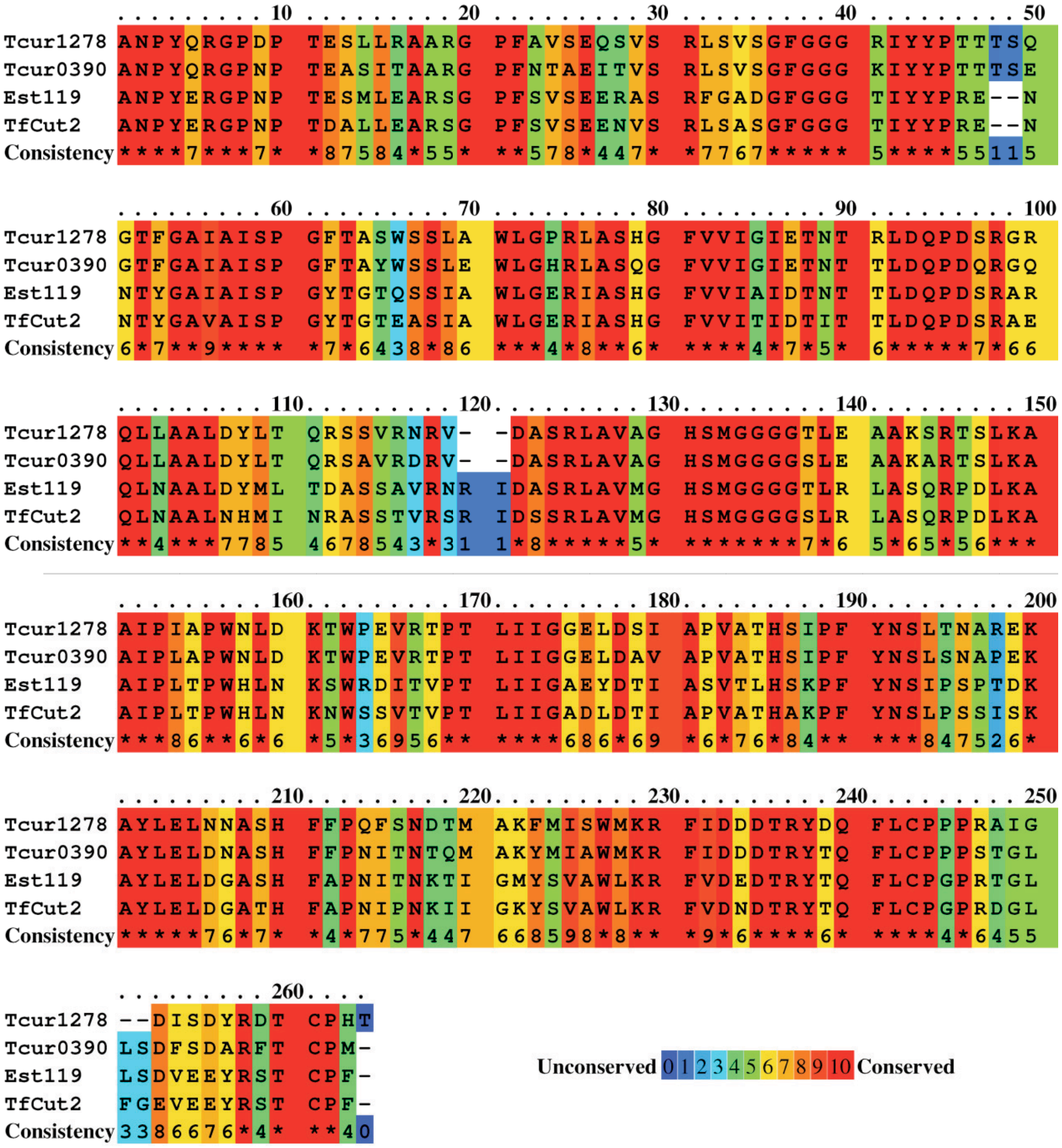

Fig. S1. Alignment of the mature protein sequences of Tcur1278, Tcur0390, TfCut2 and Est119 polyester hydrolases. The regions of similarity of individual amino acid residues are indicated with colors from blue, unconserved to red, conserved. The multiple sequence alignment was performed with the PRALINE web server (Simossis and Heringa 2005).

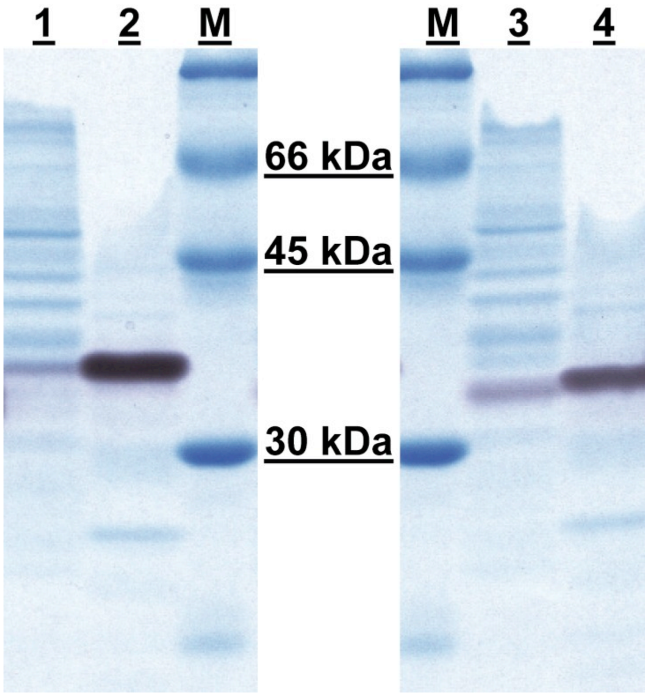

Fig. S2. SDS PAGE analysis of Tcur0390 (lanes 1-2) and Tcur1278 (lanes 3-4). 10 µg of crude cell lysate (1, 3) and eluate obtained after IMAC purification (2, 4) were loaded in each lane; protein size markers (M). The gel was first stained with Fast Red dye for esterase activity against 1-naphthyl acetate (purple bands) followed by staining with Coomassie Brilliant Blue (blue bands).
